# Clinical characteristics and risk factors for COVID-19 infection and disease severity: A nationwide observational study in Estonia

**DOI:** 10.1371/journal.pone.0270192

**Published:** 2022-06-16

**Authors:** Tatjana Meister, Heti Pisarev, Raivo Kolde, Ruth Kalda, Kadri Suija, Lili Milani, Liis Karo-Astover, Marko Piirsoo, Anneli Uusküla

**Affiliations:** 1 Institute of Family Medicine and Public Health, University of Tartu, Tartu, Estonia; 2 Institute of Computer Science, University of Tartu, Tartu, Estonia; 3 Institute of Genomics, University of Tartu, Tartu, Estonia; 4 Institute of Technology, University of Tartu, Tartu, Estonia; Taipei Medical University School of Medicine, TAIWAN

## Abstract

**Background:**

COVID-19 pandemic has led to overloading of health systems all over the world. For reliable risk stratification, knowledge on factors predisposing to SARS-CoV-2 infection and to severe COVID-19 disease course is needed for decision-making at the individual, provider, and government levels. Data to identify these factors should be easily obtainable.

**Methods and findings:**

Retrospective cohort study of nationwide e-health databases in Estonia. We used longitudinal health records from 66,295 people tested positive for SARS-CoV-2 RNA from 26 February 2020 to 28 February 2021 and 254,958 randomly selected controls from the reference population with no known history of SARS-CoV-2 infection or clinical COVID-19 diagnosis (case to control ratio 1:4) to predict risk factors of infection and severe course of COVID-19. We analysed sociodemographic and health characteristics of study participants. The SARS-CoV-2 infection risk was slightly higher among women, and was higher among those with comorbid conditions or obesity. Dementia (RRR 3.77, 95%CI 3.30⎼4.31), renal disease (RRR 1.88, 95%CI 1.56⎼2.26), and cerebrovascular disease (RRR 1.81, 95%CI 1.64⎼2.00) increased the risk of infection. Of all SARS-CoV-2 infected people, 92% had a non-severe disease course, 4.8% severe disease (requiring hospitalisation), 1.7% critical disease (needing intensive care), and 1.5% died. Male sex, increasing age and comorbid burden contributed significantly to more severe COVID-19, and the strength of association for male sex increased with the increasing severity of COVID-19 outcome. The strongest contributors to critical illness (expressed as RRR with 95% CI) were renal disease (7.71, 4.71⎼12.62), the history of previous myocardial infarction (3.54, 2.49⎼5.02) and obesity (3.56, 2.82⎼4.49). The strongest contributors to a lethal outcome were renal disease (6.48, 3.74⎼11.23), cancer (3.81, 3.06⎼4.75), liver disease (3.51, 1.36⎼9.02) and cerebrovascular disease (3.00, 2.31⎼3.89).

**Conclusions:**

We found divergent effect of age and gender on infection risk and severity of COVID-19. Age and gender did not contribute substantially to infection risk, but did so for the risk of severe disease Co-morbid health conditions, especially those affecting renin-angiotensin system, had an impact on both the risk of infection and severe disease course. Age and male sex had the most significant impact on the risk of severe COVID-19. Taking into account the role of ACE2 receptors in the pathogenesis of SARS-CoV-2 infection, as well as its modulating action on the renin-angiotensin system in cardiovascular and renal diseases, further research is needed to investigate the influence of hormonal status on ACE2 expression in different tissues, which may be the basis for the development of COVID-19 therapies.

## Introduction

On 11th March 2020, the World Health Organization (WHO) characterised COVID-19 ⎼ a condition caused by the severe acute respiratory syndrome coronavirus 2 (SARS-CoV-2) ⎼ as a pandemic [1]. The world has passed the milestone of 5 million COVID-19 deaths by October 2021 [2, 3].

Even with a background of widely implemented non-pharmacological infection containment measures (business and school closures, restrictions of movement, social distancing and total lockdowns) the COVID-19 pandemic has required unprecedented mobilisation of health systems.

The severity of COVID-19 is highly variable, ranging from asymptomatic infection to a complicated and sometimes lethal course. Case series (hospital-based studies) have identified risk factors associated with poor outcomes, including older age, male sex and certain underlying diseases [4–6].

Evidence about the first year of the COVID-19 pandemic has frequently been based on data from symptomatic patients [4–6], seroepidemiological studies [7] and modelling [8, 9]. Studies have been based on small or selected population samples (e.g. hospital admissions, or tested populations [10, 11]) providing data that is probably unrepresentative of the wider community. To the best of our knowledge, the few, large population-based studies have, so far, focused on factors related to mortality and data from COVID-19 cases only [12–15]. However, such analyses based only on COVID-19 cases are unable to cast light on the risk of acquiring the infection. Yet, primary prevention operates through the control of the true infection risk factors (both acquisition and virulence). Use of population-based data could help in delineating risk factors for SARS-CoV-2 infection and the risk of severe disease to inform patient management, public health measures and more personalised advice to patient groups [16].

The objective of this study was to examine factors associated with SARS-CoV-2 infection and the severity of COVID-19 disease in a population-based setting.

## Materials and methods

### Design

In this population-based retrospective cohort study, data on all individuals with confirmed COVID-19 were compared with randomly selected individuals from the reference group with no known history of COVID-19 prior to the index date (defined below). The data are pertinent to the first year of the COVID-19 pandemic (comprising cases from 26 February 2020 to 28 February 2021), reflecting the epidemiological situation in Estonia during the first and second waves of the disease.

The Research Ethics Committee of the University of Tartu approved our study (No. 330/T-10), and has waived the requirement to obtain any informed consent.

### Data sources

#### National communicable disease case notification data

The Estonian Health Board (EHB) has a national case-based surveillance system and is the state agency responsible for communicable disease surveillance, national and local epidemiological services and implementation of the national immunization scheme [17]. The EHB collects and analyses notification data on communicable diseases (which now includes all SARS-CoV-2 testings). All confirmed COVID-19 cases are based on real-time polymerase chain reaction (PCR) testing on nasopharyngeal specimens by a certified molecular diagnostics laboratory using certified methods [18].

#### The Estonian Health Insurance Fund (EHIF)

By the end of 2020, universal public health insurance covered 95.2% of the Estonian population (1,328,889 people) [19, 20]. Since its inception in the early 2000s, the Estonian Health Insurance Fund (EHIF) has maintained a complete record of health care services [21]. The EHIF electronic administrative database contains personal information (sex, age), health care utilisation (date of service, primary and other diagnoses, treatment type (in- or out-patient), types of services provided), and the date of death. In addition to data on the individual receiving care and the care provider, health care claims contain diagnostic codes based on the International Classification of Diseases, tenth revision (ICD-10), and all medical services and procedures provided (NOMESCO [22], EHIF-specific procedure codes [23]).

It is important to note that since the beginning of the pandemic all residents, regardless of their insurance and employment status, had access to SARS-CoV-2 PCR testing and, if needed, were provided with outpatient or hospital treatment, including critical care [24].

#### Estonian causes of death registry

The Causes of Death Registry collects data on all deaths registered in Estonia. Deaths are reported to the registry using a standardized death certificate including the date of death [25].

### Study population

#### COVID-19 group (cases)

COVID-19 cases were identified from the national passive surveillance database. Data on confirmed COVID-19 cases (national personal identification code, the date of SARS-CoV-2 specimen collection, and testing) were retrieved from the EHB. The index date of COVID-19 diagnosis cases was defined as the date of the first SARS-Cov-2 RNA positive specimen collection. We included all confirmed COVID-19 cases for the period 26 February 2020 to 28 February 2021 in Estonia.

#### Reference (control) group

Cases were matched to a random subset of individuals insured by the EHIF who at COVID-19 case index date were alive and had no evidence of COVID-19 using a case to control ratio of 1:4 [26].

For both the COVID-19 cases and the reference population, health data was abstracted from the EHIF database for the period of 24 months before, and up to 2 months after, the index date.

### Outcome and covariates

#### Identification of COVID-19 severity

For the analysis, we defined ‘acute COVID-19’ as a period extending from the two weeks before, up to 30 days after, the index date [27].

We defined COVID-19 disease severity as follows: (i) non-severe disease (patients requiring no medical care or ambulatory medical care only); (ii) severe disease (patients requiring hospitalisation but no intensive care); (iii) critical disease (patients receiving intensive care) during the acute COVID-19 period; and, (iv) lethal disease (patients who died during the acute COVID-19 period) [28]. We ascertained how many individuals with COVID-19 had non-severe, severe and critical COVID-19 disease or died during the acute COVID-19 period.

#### Identification of pre-COVID-19 comorbidity

Comorbidities were captured for the 365-day period prior to the index date for COVID-19 patients and controls. The comorbidity status for both groups was computed using the Charlson Comorbidity index (CCI) [29]. The index is based on the hazard ratios of individual life-threatening comorbidities for 1-year mortality presented as disease weights, and the CCI score represents the person’s mortality-predicting disease burden. In addition to CCI component diseases, we also included co-morbidities and health states of interest such as obesity (defined as ICD-10 codes E66.0⎼E66.9) [30, 31], hyperlipidaemia (based on ICD-10 codes E78.0⎼E78.9) [32], having influenza or influenza-like illness, or vaccination against influenza [33–35]. Comorbidities were identified and defined by the ICD-10 code occurring at least once on inpatient health care claims or twice occurring over more than 30 days on outpatient health care claims during the year preceding the index date.

Episodes of or influenza-like illness and vaccination against influenza (occurring at least 30 days prior to the index date) were defined from ICD-10 codes (J00⎼J01, J04⎼J06, J09⎼J12, J20⎼J22, J02.9, J03.9, J17.1, and Z25.1, respectively) and assessed for one and two years before a positive SARS-CoV-2 PCR test.

### Statistical analysis

Demographic and health status data are reported as frequencies and proportions for categorical variables and as median and standard deviation (SD) for continuous variables.

To identify COVID-19 risk factors, we used conditional logistic regression to demonstrate differences between COVID-19 cases and reference group individuals, with results presented as adjusted OR and 95% confidence intervals (CI) (adjusted for age, gender and comorbidity). We used the revised coding algorithm described by Quan, et al. [36], subsequently validated for estimating comorbidity using ICD-10 coded administrative data [37, 38]. The CCI consists of 14 comorbidities (CCI components). Comorbidities are weighted from 1 to 6 for mortality risk and then summed to form the total CCI score (groups ranging 0, 1–2 and ≥3).

To identify factors associated with COVID-19 disease severity among SARS-CoV-2 positive cases, we present proportions for categorical variables and median and SD for continuous variables. To compare COVID-19 disease severity groups, we used chi-squared test or Kruskal-Wallis test, as appropriate (S1 Table). A multinomial logistic regression model adjusted for age, gender and Charlson index was also computed to produce adjusted relative risk ratios (RRR), together with 95% CIs for the comparisons (i.e. severe, critical, and lethal COVID-19 disease versus non-severe).

In our sample, individuals in the reference population (controls) were at risk of SARS-CoV-2 infection. The SARS-CoV-2 incidence rate was calculated by computing the infection-free observation time for each control, adding up the disease-free observation times for the entire control group, and then dividing this into the number of SARS-CoV-2 cases. We present incidence rate per 10,000 follow-up months with the 95% confidence intervals.

All analyses were performed in Stata 14.2 with an additional Charlson macro (for Charlson index calculations).

## Results

### Study cohorts

From 26 February 2020 to 28 February 2021, 66,295 people tested positive for SARS-CoV-2 RNA in Estonia (5% of the total population) [39]. Their data, and data on the randomly selected 254,958 individuals from the reference population, are presented in Table 1. People with SARS-CoV-2 infection were slightly older, with a mean age of 44.1 (SD 20.6) years in the COVID-19 group and 42.3 (SD 22.9) years in the control group (a difference of 1.8 years, 95% CI 1.5–1.9 years, p<0.001); 35,808 (54.01%) and 130,600 (51.22%) of subjects were female (p<0.001), in the COVID-19 and reference groups, respectively.

**Table 1:** Characteristics of patients with COVID-19 and controls in Estonia for the period of 26 February 2020 to 28 February 2021.

	SARS-CoV-2 infection n = 66295		Reference group n = 254958		Adjusted odds ratio (95% CI)	p-value
	n	%	n	%		
***Sociodemographic characteristics***						
*Women*	35808	54.01	130600	51.22	1.1 (1.08–1.12)	p<0.001 ^1^
*Age*, *mean (sd)*	44.1 (20.6)		42.3 (22.9)		1.02 (1.02–1.03)	p<0.001 ^2^
*Age groups (years)*						
Under 50	40626	61.28	158827	62.3	1	
50⎼59	10096	15.23	32580	12.78	1.18 (1.15–1.21)	p<0.001
60⎼69	8253	12.45	29469	11.56	1.02 (0.99–1.05)	p = 0.127
70⎼79	3818	5.76	20048	7.86	0.66 (0.63–0.68)	p<0.001
80 years and older	3502	5.28	14034	5.5	0.84 (0.8–0.87)	p<0.001
*Age groups (years)*						
0⎼9 years	2289	3.45	24584	9.64	0.34 (0.32⎼0.35)	p<0.001
10⎼19	7219	10.89	25962	10.18	1.01 (0.98⎼1.05)	P = 0.452
20⎼29	8226	12.41	28286	11.09	1.06 (1.03⎼1.1)	p<0.001
30⎼39	11704	17.65	42522	16.68	1	
40⎼49	11188	16.88	37473	14.7	1.08 (1.05⎼1.11)	p<0.001
50⎼59	10096	15.23	32580	12.78	1.1 (1.06⎼1.13)	p<0.001
60⎼69	8253	12.45	29469	11.56	0.95 (0.92⎼0.98)	P = 0.002
70⎼79	3818	5.76	20048	7.86	0.61 (0.59⎼0.64)	p<0.001
80 and older	3502	5.28	14034	5.5	0.78 (0.74⎼0.81)	p<0.001
***Pre-COVID-19 comorbitity*** ^**3**^						
Charlson index score, mean (sd)	0.15 (0.52)		0.11 (0.43)		1.19 (1.17–1.21)	p<0.001 ^3^
*Charlson index score range*	0⎼9		0⎼9			
*Charlson index score groups*						
0	59536	89.8	236288	92.68	1	
1–2	4246	6.4	12226	4.8	1.31 (1.26–1.36)	p<0.001 ^3^
3 or more	2513	3.79	6444	2.53	1.47 (1.40–1.54)	p<0.001
*Charlson Index components*						
Acute Myocardial Infarction	299	0.45	940	0.37	1.15 (1.01–1.31)	p = 0.041 ^3^
Congestive heart failure	1544	2.33	4406	1.73	1.22 (1.15–1.29)	p<0.001
Peripheral Vascular Disease	202	0.3	612	0.24	1.17 (1–1.38)	p = 0.052
Cerebrovascular Disease	612	0.92	1196	0.47	1.81 (1.64–2)	p<0.001
Dementia	469	0.71	426	0.17	3.77 (3.3–4.31)	p<0.001
Chronic pulmonary disease	1063	1.6	3200	1.26	1.24 (1.16–1.33)	p<0.001
Rheumatologic disease	356	0.54	1270	0.5	0.98 (0.87–1.1)	p = 0.703
Peptic Ulcer	139	0.21	398	0.16	1.27 (1.05–1.54)	p = 0.016
Chronic kidney disease	171	0.26	322	0.13	1.88 (1.56–2.26)	p<0.001
AIDS/HIV	15	0.02	37	0.01	1.58 (0.86–2.88)	p = 0.138
Diabetes	1485	2.24	4065	1.59	1.33 (1.25–1.41)	p<0.001
Cancer	1305	1.97	3698	1.45	1.27 (1.19–1.35)	p<0.001
Liver Disease	185	0.28	437	0.17	1.58 (1.33–1.87)	p<0.001
***Other comorbidities***						
Obesity	1163	1.75	2474	0.97	1.61 (1.5–1.73)	p<0.001
Hyperlipidaemia	2287	3.45	7058	2.77	1.11 (1.05–1.16)	p<0.001
Hypertension	12168	18.35	37257	14.61	1.19 (1.16–1.23)	p<0.001
***Influenza and vaccination within 2 years before COVID-19***						
Influenza (during last 2 years)	20863	31.47	62127	24.37	1.48 (1.45–1.51)	p<0.001 ^4^
Flu vaccine (during last 2 years)	5083	7.67	15551	6.1	1.21 (1.17–1.25)	p<0.001

^1^ Odds ratio adjusted to gender and Charlson score;

^2^ Odds ratio adjusted to age and Charlson score;

^3^ Odds ratio adjusted to gender and age;

^4^ Odds ratio adjusted to age, gender and Charlson score

Both cohorts were relatively healthy: 89.8% of COVID-19 cases and 92.68% of reference cohort individuals scored 0 on the CCI Score. In both groups, hypertension, hyperlipidaemia, congestive heart failure, and diabetes were the most common co-morbid conditions. However, there were significant differences in the proportion of affected individuals by group. The most common co-morbid disease, hypertension, occurred in 18.35% of COVID-19 cases and 14.61% of individuals in the reference population (p<0.001); hyperlipidaemia (3.45% and 2.77%, COVID-19 cases and the reference population respectively, p<0.001); congestive heart failure (2.33% and 1.73%, p<0.001); and diabetes (2.24% and 1.59%, p<0.001).

During the study period, 6032 individuals from the control group tested positive for SARS-CoV-2 RNA, giving an incidence of 116.99 (95%CI 114.08–119.98) per 10,000 person-months.

### Factors associated with COVID-19 infection

**Fig 1:**
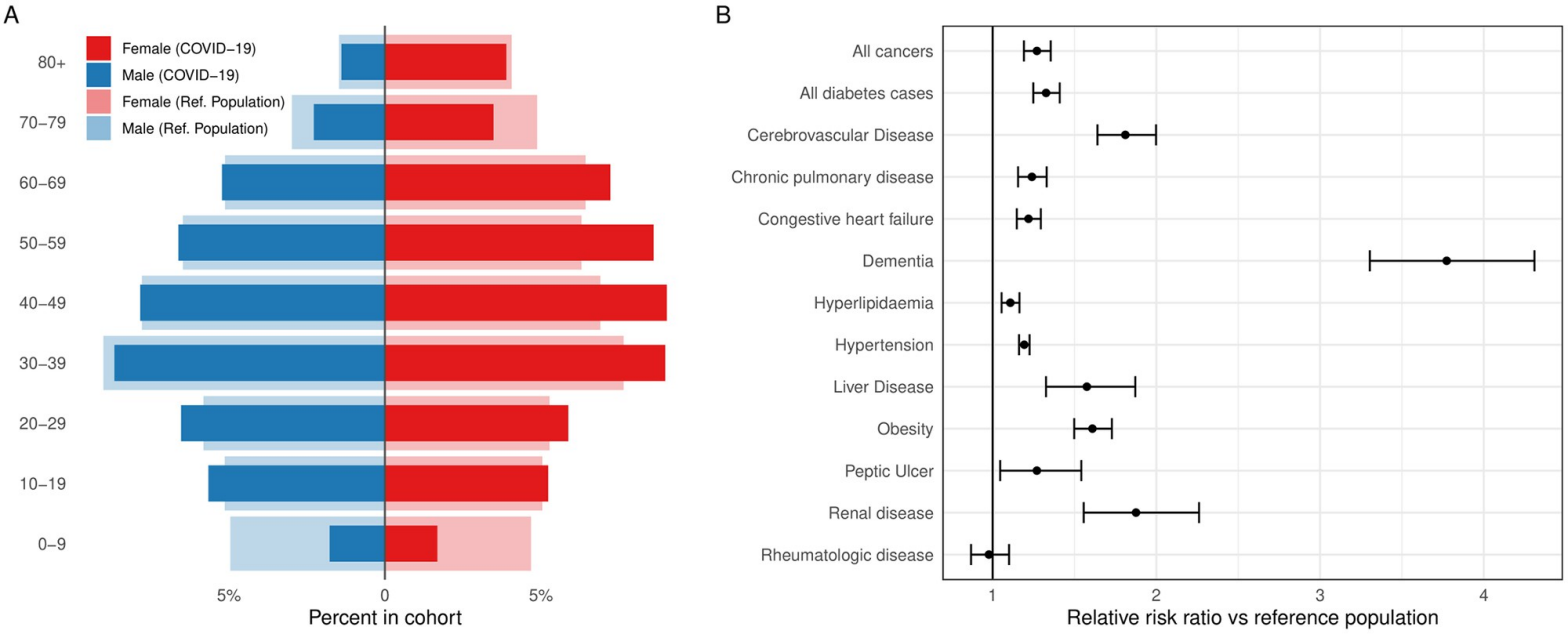
Factors associated with COVID-19 infection. (A) Proportion of patients with positive SARS-CoV-2 PCR test stratified by age and sex compared with the general population. (B) Forest plot showing the association between co-morbid diseases and risk of SARS-CoV-2 infection, adjusted for age and sex.

In multivariable conditional logistic regression analysis, women were slightly more likely to have a positive SARS-CoV-2 test than men (OR 1.1 (95% CI 1.08–1.12)). We found a very weak effect of age on the risk of acquiring SARS-CoV-2 infection (for ten years OR 1.02 (1.02–1.03)). Children (< 9 years) and elderly people (aged 60+ years) had lower odds for infection than adults. Fig 1A shows a histogram of the percentages of SARS-CoV-2 positive individuals and controls by sex and 10-year age interval.

Compared to a CCI score of 0, a CCI score of 1–2 was associated with a higher risk of being infected with SARS-CoV-2 (OR 1.31, 95% 1.26⎼1.36) and the strength of the effect increased with the index score increasing, rising to 47% in those with CCI 3 or above (OR 1.47, 95% CI 1.4⎼1.54).

COVID-19 cases had a significantly higher prevalence of dementia (OR 3.77, 95% CI 3.3⎼4.31); renal disease (OR 1.88, 95% CI 1.56⎼2.26); cerebrovascular disease (OR 1.81, 95% CI 1.64⎼2); liver disease (OR 1.58, 95% CI 1.33⎼1.87); diabetes (OR 1.33, 95% CI 1.25⎼1.41); cancer (OR 1.27, 95% CI 1.19⎼1.35); chronic pulmonary disease (OR 1.24, 95% CI 1.16⎼1.33); and congestive heart failure (OR 1.22, 95% CI 1.15⎼1.29) (Fig 1B). Obese individuals had higher odds for COVID-19 infection (OR 1.61, 95% CI 1.5⎼1.73) as had individuals with hypertension (OR 1.19, 95% CI 1.16⎼1.23) or hyperlipidaemia (OR 1.11, 95% CI 1.05⎼1.16) (see Table 1).

### COVID-19 disease severity

Among the 66,142 patients with a positive SARS-CoV-2 PCR test, the overwhelming majority (92%, n = 60,845) had a non-severe course of COVID-19, however, 4.8% (n = 3,198) had severe disease (required hospitalisation without intensive care), 1.7% (n = 1,129) had critical disease (needing intensive care), and 1.5% (n = 970) died within 30 days of being tested positive for SARS-CoV-2 (S1 Table).

#### Non-severe COVID-19

Approximately half of the individuals with non-severe disease were women (54%), the mean age was 42.0 (range 0–103.7) years, with two-thirds (65.21%) being younger than 50 years of age, and only 7.74% of cases in this group had one or more co-morbid diseases.

#### Severe COVID-19

Among those COVID-19 cases hospitalised but not needing intensive care, 57.44% were women, the mean age was 62.9 years (range 0–103.7), with a quarter (23.7%) being younger than 50 years of age; one-third (30%) had a co-morbid condition in addition to COVID-19.

#### Critical disease

COVID-19 patients in need of intensive care were, on average 67.3 (range 0–100.5) years old, less than half were women (44.2%), with just over one-tenth (12.67%) under 50 years old and 41% had co-morbidities.

Among those patients hospitalised due to COVID-19 (n = 4,327), the length of hospital stay was twice as long as for those admitted to a critical care unit (median 17 days versus 8 days, p<0.001). Patients dying in the acute Covid-19 period had the shortest hospital stay (a median of 6 days).

#### Lethal disease

Of all SARS-CoV-2 positive individuals, the 1.5% who died during acute COVID-19 were on average 80.9 (range 0–103.5) years old. Dying at an age of less than 50 years was exceedingly rare (1.44% of all deaths). Two-thirds of the deaths occurred in the group aged 80 years or more; more than half (58.25%) had one or more co-morbid conditions.

### Factors associated with severity of COVID-19

In the multinomial logistic regression analysis, increasing age and pre-existing chronic renal, cardiovascular and metabolic diseases were strongly associated with higher risk of severe COVID-19. Meanwhile, female sex was clearly associated with lower risk of severe COVID-19.

**Fig 2:**
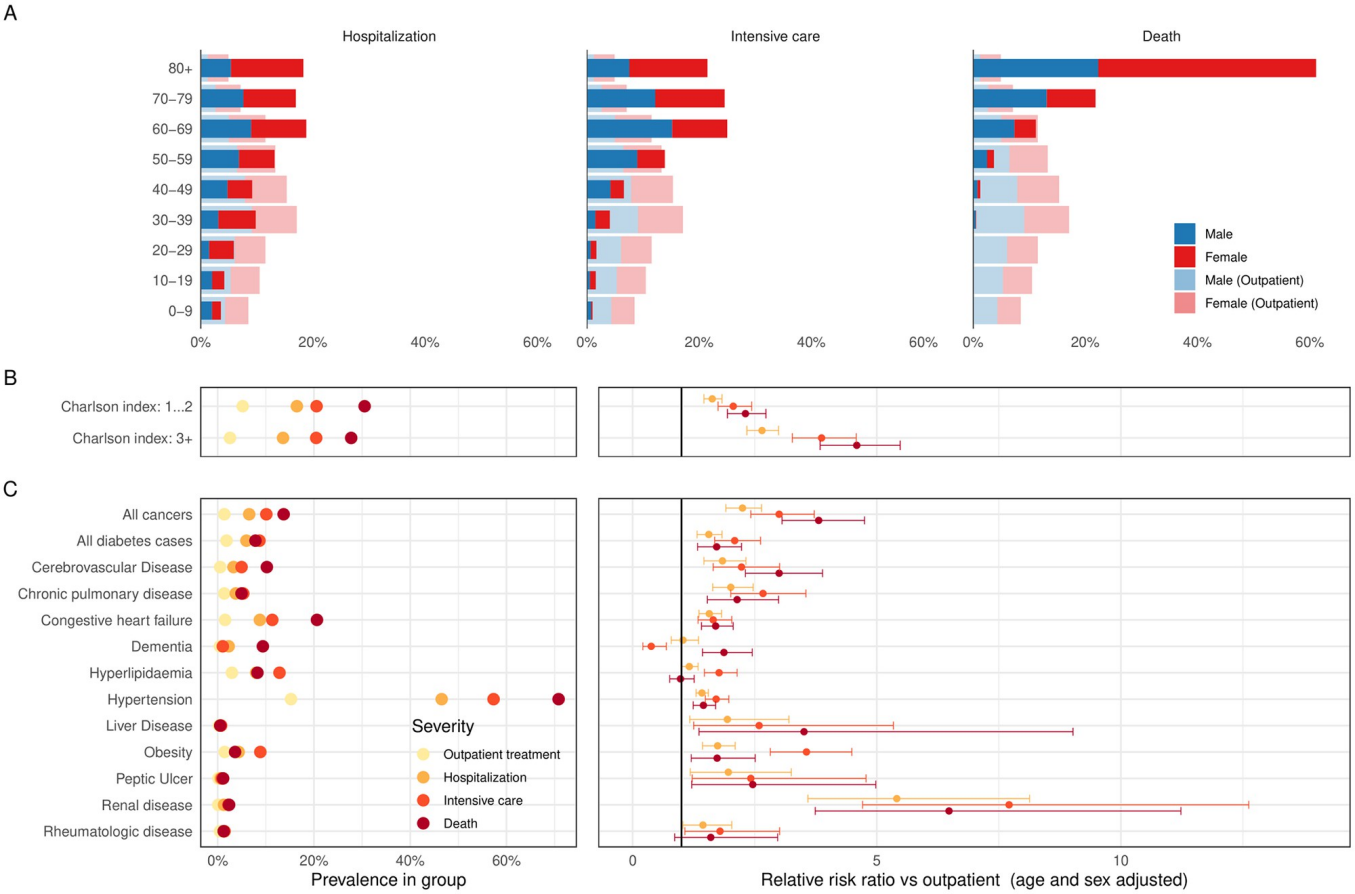
Factors associated with COVID-19 severity. (A) Proportion of patients with severe COVID-19 disease (hospitalized, admitted to intensive care unit or died) stratified by age and sex compared with those who required outpatient treatment only. (B) Forest plot showing the association between CCI index and risk of COVID-19 associated hospitalization, intensive care treatment and death, adjusted for age and sex. (C) Forest plot showing the association between comorbid diseases and risk of COVID-19 associated hospitalization, intensive care treatment and death, adjusted for age and sex.

Age had the strongest effect on the severity of COVID-19: a 10-year increase in age was associated with a RRR of 1.68 (95% CI 1.65⎼1.72) for severe disease and RRR of 3.91 (95% CI 3.7⎼4.19) for lethal disease compared to non-severe disease. In comparison to those aged <50 years, individuals aged 50⎼59 years were twice as likely to develop severe disease (RRR 2.43, 95% CI 2.16⎼2.73), four-times more likely (RRR 4.46, 95% CI 3.55⎼5.6) to require intensive care treatment and almost 9 times more likely to die (RRR 8.61, 95% CI 4.56⎼16.25). For those aged 80 and above, there was a 16-fold risk (RRR 16.55, 95% CI 14.69⎼18.65) of developing severe disease and an extremely high risk of a fatal outcome (RRR 784.11 95% CI 485.38⎼1341.3). Fig 2A shows histograms of the percentages of severe, critical and lethal COVID-19 cases in compare to non-severe COVID-19 cases by sex and 10-year age interval.

The protective effect of female sex was strong and the strength of association increased with diseases severity (from RRR 0.84, 95% CI 0.78–0.91 for severe COVID-19 to 0.41 95% CI 0.35–0.47 for lethal COVID-19).

Compared to a CCI score of 0, a CCI score of 1 or more was associated with a higher risk of severe disease outcome (hospitalisation RRR 1.63, 95% CI 1.46⎼1.83 for CCI of 1⎼2), and the strength of the effect increased with the index score increasing (hospitalisation RRR 2.65, 95% CI 2.34⎼2.99 for CCI of 3 or more) (Fig 2B).

The presence of renal disease (lethal disease RRR 6.48, 95% CI 3.74⎼11.23), liver disease (lethal disease RRR 3.51, 95% CI 1.36⎼9.02), any type of cancer (lethal disease RRR 3.81, 95% CI 3.06⎼4.75), and cerebrovascular disease (lethal disease RRR 3.00, 95% CI 2.31⎼3.89) concomitant to SARS-CoV-2 infection had the strongest association with COVID-19 disease severity (Fig 2C).

Other chronic conditions associated with a three-fold increase in risk of a severe course of COVID-19 were history of previous myocardial infarction (critical care RRR 3.54, 95% CI 2.49⎼5.02), obesity (critical care RRR 3.56, 95% CI 2.82⎼4.49) and peripheral vascular disease (critical care RRR 3.38, 95% CI 2.21⎼5.18) (see Table 2).

**Table 2:** Multivariable analysis of patient characteristics associated with acute COVID-19 severity in Estonia for the period of 26 February 2020 to 28 February 2021.

	Severe disease		Critical disease		Lethal disease	
	RRR	(95% CI)	RRR	(95% CI)	RRR	(95% CI)
**Sociodemographic characteristics**						
*Gender (women)*	0.84***	[0.78⎼0.91]	0.44***	[0.39⎼0.50]	0.41***	[0.35⎼0.47]
Age, *10yr change*	1.68***	[1.65⎼1.72]	1.97***	[1.90⎼2.05]	3.91***	[3.70⎼4.19]
*Age groups*						
Under 50	1		1		1	
50–59	2.43***	[2.16⎼2.73]	4.46***	[3.55⎼5.6]	8.61***	[4.56⎼16.25]
60–69	4.43***	[3.97⎼4.94]	10.05***	[8.2⎼12.33]	34.84***	[19.89⎼61.04]
70–79	9.51***	[8.44⎼10.71]	22.62***	[18.29⎼27.99]	172.98***	[100.26⎼298.46]
80 and older	16.55***	[14.69⎼18.65]	33.60***	[26.95⎼41.88]	784.11***	[485.38⎼1341.3]
**Pre-COVID-19 comorbitity**						
*Charlson index score*	1.51***	[1.44⎼1.59]	1.73***	[1.63⎼1.84]	1.83***	[1.71,1.95]
*Charlson index score groups*						
1–2	1.63***	[1.46⎼1.83]	2.06***	[1.75⎼2.44]	2.31***	[1.94⎼2.73]
3 or more	2.65***	[2.34⎼2.99]	3.87***	[3.27⎼4.58]	4.59***	[3.84⎼5.48]
Acute Myocardial Infarction	1.67**	[1.19⎼2.34]	3.54***	[2.49⎼5.02]	1.56	[0.98⎼2.49]
Congestive heart failure	1.57***	[1.36⎼1.82]	1.65***	[1.34⎼2.03]	1.7***	[1.43⎼2.06]
Peripheral Vascular Disease	2.38***	[1.66⎼3.43]	3.38***	[2.21⎼5.18]	1.73*	[1.04,2.90]
Cerebrovascular Disease	1.84***	[1.46⎼2.32]	2.23***	[1.65⎼3.01]	3.00***	[2.31⎼3.89]
Dementia	1.03	[0.79–1.35]	0.38**	[0.21⎼0.69]	1.85***	[1.43⎼2.45]
Chronic pulmonary disease	2.01***	[1.64⎼2.47]	2.67***	[2.01⎼3.55]	2.14***	[1.53⎼2.99]
Rheumatologic disease	1.44*	[1.03⎼2.03]	1.79*	[1.07⎼3.01]	1.6	[0.86⎼2.97]
Peptic Ulcer	1.96**	[1.18⎼3.25]	2.42*	[1.22⎼4.78]	2.46*	[1.21⎼4.98]
Chronic kidney disease	5.41***	[3.59⎼8.13]	7.71***	[4.71⎼12.62]	6.48***	[3.74⎼11.23]
AIDS	5.01*	[1.08⎼23.12]	16.77***	[3.53⎼79.79]	0	[0.00]
Diabetes	1.56***	[1.32⎼1.83]	2.09***	[1.68,2.62]	1.72***	[1.33⎼2.23]
Cancer	2.25***	[1.91⎼2.64]	3.00***	[2.42,3.72]	3.81***	[3.06⎼4.75]
Liver Disease	1.94**	[1.17⎼3.2]	2.59**	[1.25⎼5.34]	3.51***	[1.36⎼9.02]
*Other comorbidities*						
Obesity	1.74***	[1.43⎼2.10]	3.56***	[2.82⎼4.49]	1.73**	[1.2⎼2.51]
Sleep apnea	1.71**	[1.16⎼2.52]	2.54***	[1.55⎼4.16]	1.45	[0.67⎼3.12]
Hyperlipidaemia	1.16*	[1.01⎼1.34]	1.77***	[1.47⎼2.14]	0.98	[0.76⎼1.26]
Hypertension	1.42***	[1.3⎼1.55]	1.71***	[1.49⎼1.97]	1.45***	[1.24⎼1.70]
**Influenza and vaccination within 2 years before COVID-19**						
Flu vaccine (during last 2 years)	1.08	[0.96⎼1.20]	0.85	[0.7⎼1.02]	0.88	[0.74⎼1.06]
Influenza (during last 2 years)	1.22***	[1.13⎼1.33]	1.14	[0.99⎼1.32]	0.92	[0.76⎼1.11]

* p<0.05,

** p<0.01,

*** p<0.001;

RRR is found in comparison to the non-severe COVID-19

With did not see association between seasonal flu vaccine administrated within the past two years before COVID-19 either with the critical course (RRR 0.85, 95% CI 0.7⎼1.02) or lethal (RRR 0.88, 95% CI 0.74⎼1.06) course of the SARS-CoV-2 infection. Flu, or influenza-like disease, during the last two year prior to infection increased the risk of a severe course of COVID-19 leading to hospitalisation (RRR 1.22, 95% CI 1.13⎼1.33) and treatment in an intensive care unit RRR 1.14, 95% CI 0.99⎼1.32).

## Discussion

We evaluated nationwide data of people who tested positive for SARS-CoV-2 RNA during the first year of the pandemic in Estonia (representing the period before a COVID-19 vaccination was available), and compared it with that of people from the general population. To our knowledge, this is one of the largest clinical studies assessing factors associated with SARS-CoV-2 infection and the severity of COVID-19 disease in a population-based setting.

### SARS-CoV-2 infection risk factors

Previously, older age and male gender have been reported as important independent predictors of SARS-CoV-2 infection [10, 11], but not consistently. Some previous studies showed that the rate of SARS-CoV-2 infection (confirmed by positive PCR test or antibody testing) is comparatively the same [40, 41] or even higher [42] in adolescents than in adults, and that women may be at greater risk of infection than men [43]. Our data suggest that women were at slightly higher risk for SARS-CoV-2 infection. This result is somewhat conflicting with the estimate from a recent review, in which men were found to be at slightly higher (8%) risk of COVID-19 [11]. Yet, this review was overwhelmingly based on studies reporting experiences of clinical (ie hospitalised) cohorts. A study from the UK, synthesising data originating from a primary care sentinel network, documented even higher risk of males (compared to women) for SARS-CoV-2 infection [10]. However, the authors mention that testing initially focused on people who travelled to high-risk countries or close contact tracing. Estonia has been one of the countries with the highest COVID-19 testing rates (totally, 708 SARS-CoV-2 PCR tests per 1000 people by 28 February, 2020) [44] and symptomatic or close contact-driven testing was replaced with more population-based testing early in the course of the epidemic [45]. We acknowledge that the results we are reporting here might be influenced by the tendency among women to make greater use of health care services [46]. However, we would still speculate that the sex differences for the risk of contracting SARS-CoV-2 are smaller than suspected so far.

In addition, we saw a marginal to non-significant effect of age on the risk of SARS-CoV-2 infection, except for people in the 50–59 age group for whom we found a moderate increase in risk. We acknowledge that lower infection risk among children in our analysis might be related to lower testing rates among children potentially exposed or symptomatic [47].

Our analysis confirmed that individuals with preceding health problems are at increased risk of SARS-CoV-2 infection. We saw the strongest effect of increasing risk from dementia, renal disease, cerebrovascular disease and obesity. In general, the effect of other pre-existing health conditions on the risk of SARS-Cov-2 infection, such as cancer or chronic lung disease, was weak. We did not find an association between rheumatic disorders and the risk of SARS-CoV-2 infection. Dementia had the strongest association with COVID-19, increasing the risk of being tested positive for SARS-CoV-2 by almost four times. We are aware that general testing conducted in care homes in Estonia early in pandemic could impact the results, leading to a possible overestimation of the true effect [45]. But these findings are still in concordance with other similar studies, revealing the association between chronic cerebrovascular, cardiovascular [48] and psychiatric conditions [49] (including dementia) [50] on the risk of SARS-CoV-2 infection. Therefore, there is reason to believe that association between dementia and the risk of SARS-CoV-2 infection is not accidental and is not simply due to confounding by older age and comorbid conditions.

### Course of COVID-19 and factors associated with the severity of the disease

Our findings that men, and individuals with co-morbid conditions, are at higher risk for severe COVID-19, are echoed by many other studies [43, 48, 51, 52]. In Estonia, SARS-CoV-2 infected individuals with cardiovascular disease, cerebrovascular disease, chronic pulmonary diseases, peptic ulcer, diabetes, cancer, liver disease or obesity, were at increased risk of a severe outcome (after adjusting for age and gender), doubling or tripling the risk of a critical COVID-19 course compared with people without such conditions. These findings are consistent with those of previous reports, highlighting the significant role of comorbidities and primarily of cardiovascular disease as a risk factor for developing severe or fatal acute COVID-19 [30, 31].

Hypertension has relatively modest effect on the risk of severe COVID-19, but is the most common co-morbid condition, therefore it’s contribution at the population level is likely to be substantial (Fig 2C).

Renal disease, including hypertensive renal failure, had the strongest impact on the risk of a severe course of COVID-19 and lethal outcome. Studies show that ACE2 (angiotensin converting enzyme 2)–the enzyme that protects the cardiovascular system from the action of angiotensin II—is altered in diabetes [53, 54] and hypertensive renal disease [55]. Moreover, SARS-CoV-2 infection causes ACE2 down-regulation through binding the virus to ACE2 receptors and thus contributing to inflammatory cytokine release and clinical deterioration [56].

Some details warrant attention. In our study, we found a negative association between dementia and the risk of being admitted to hospital due to COVID-19. Moreover, multinomial regression analysis revealed a negative association between dementia and hospital or intensive care treatment admission risk (after adjusting for age and gender). Despite this, dementia was associated with an almost two-fold increase in risk of death. This observation makes us doubt whether people with cognitive impairment, especially those living in care homes, have access to appropriate health care on an equal basis with people without cognitive impairments. An analysis of health care utilization in Estonia conducted in 2015 showed that patients with dementia were less likely to receive planned medical care, as demonstrated by their lower number of outpatient visits, lower number of preventative consultations for cardiovascular disease and a higher number of avoidable hospital admissions [57]. Another study found that people with dementia were less likely to receive the services they need, and that they often experience difficulty accessing home- and community-based services [58].

A study from the USA, using electronic health records from over 70,000 individuals, outlined the potential protective effect of an influenza vaccination in SARS-CoV-2-positive patients against adverse outcomes extending over the acute COVID-19 period [59]. In our analysis, this protective effect was not observed (even though there was a tendency towards protection from most severe course of COVID-19). We might speculate that decreased risk for COVID-19-associated hospitalization among people vaccinated against influenza stems from more conscious approach of those people toward their health, leading to better control on comorbidities [60–62]. Further research is needed on the topic of treatment adherence in chronic diseases and COVID-19 outcomes.

The relevance of assessing infectivity (infection risk) and virulence (the severity of the illness) factors separately is not limited to pre-clinical, virological research [63]. Understanding characteristics of individuals associated with a higher infection acquisition risk, or more severe disease course, are vital for informing and promoting prevention and treatment efforts.

For SARS-CoV-2, and COVID-19, there is a sizeable overlap in infection risk and disease severity predictors. The pre-infection health status affecting both is quite convincing. For example, having a preceding pathology in which renin–angiotensin system (RAS) and vascular endothelium damage (i.e. chronic renal, lung, cardiovascular, cerebrovascular or peripheral vascular diseases) plays a significant role in both.

For some factors, the risk of infection and the risk of developing severe disease did not coincide. There seems to be a divergence of effects of age and gender. Both appear to be less important in driving infection risk than other factors, but quite significant for the course of COVID-19 disease. A similar pattern was observed for patients with rheumatic disease, which appeared to have no impact on risk of infection, but was associated with a significant increase in the risk of severe or critical COVID-19. For many infectious diseases (polio, measles, smallpox, HIV, MERS-CoV), children have the least severe disease, and severity increases with age (for some diseases long before old age) [64]. COVID-19 is not an exception here. Apart from potential differences in exposure and transmission rates, biological differences in the immune system exists between women and men and results in distinct manifestations of infectious diseases [65, 66]. In the case of COVID-19, gender seems to have more effect on the course of the disease. In the case of several infectious diseases (parasitic diseases [67], tuberculosis [68], HIV [69]), men are more severely affected. Yet, the opposite is true for influenza [70, 71]. Taking into account the role of ACE2 receptors in the pathogenesis of SARS-CoV-2 infection [72], as well as its protective and modulating action on the renin-angiotensin system in cardiovascular and renal diseases [73], further research is needed to investigate the influence of sex and hormonal status on ACE2 expression in different tissues which may be a gateway for virus entrance.

### Limitations and strengths

Some limitations in our research should be considered. We are aware of the potential misclassification of controls (due to people having undiagnosed SARS-CoV-2 infection). One might speculate that this misclassification is rather limited (owing to the very low prevalence of undiagnosed SARS-CoV-2 infection in the population) [74, 75] but it could lead to underestimating the true effect of infection risk factors (producing more conservative estimates).

A further limitation is the definition of co-morbid states based on administrative health data. However, we used a comorbidity index (CCI) that is rigorously tested and validated for estimating comorbidity using ICD-10 coded administrative data [37] uniformly for both comparison groups. We were limited to using ICD-10 codes to define health states of interest (obesity and hyperlipidaemia), knowing this to be suboptimal compared to direct measurement of weight and serum lipid levels. Yet, we argue that this would lead to an underestimation of the states in both comparison groups, potentially underestimating the real effect. Our data covers the pre Covid-19 vaccination period. The results described here might not be fully applicable for post-vaccination Covid-19 [76].

We were not able to control for potential confounders (i.e. exposure to COVID-19 cases, health behaviour, and the socio-economic status of people). This is a weakness of our work. However, we were able to control for known confounding factors such as age, gender and comorbidity.

The strength of our analysis lies in the use of population-based data that allows the generalisation of the results and is free of individual recall/social desirability bias. Our study has the benefits of a large cohort, allowing the estimation of weak effects. The data sources used (EHIF and Health Board) both have national coverage, and the potential bias arising from individuals`s insurance status was precluded by free access to SARS-CoV-2 PCR testing and subsequent treatment. Our case definition is reliable (based on testing results), and measurements of disease states (hospitalised, received intensive care, deceased) are robust and reliable.

## Conclusions

Our results contribute to the knowledge of how sex, age, and various chronic diseases influence the risk of infection and severe disease. We found, that the risk of SARS-CoV-2 infection is independent of age and sex, however older age, male sex and selected concomitant diseases (especially those affecting vascular endothelium of different organs) were associated with severe disease course. Multiple concomitant chronic health conditions contributed to both the risk of infection and the risk of severe disease.

## Supporting information

S1 Table Characteristics of 66,295 patients with COVID-19 in Estonia for the period of 26 February 2020 to 28 February 2021 by stage of disease severity. (DOCX)
