# Supplementary material for: Clinical characteristics and risk factors for COVID-19 infection and disease severity: A nationwide observational study in Estonia
Source: PLoS One. 2022 Jun 16;17(6):e0270192. doi: 10.1371/journal.pone.0270192 (PMC9202832; doi:10.1371/journal.pone.0270192)
Supplement: S1 Table — (DOCX) [file pone.0270192.s001.docx]

**S1 Table. Characteristics of  66,295 patients with COVID-19  in Estonia for the period of 26 February  2020 to 28 February 2021 by stage of disease severity.^[[1]](#endnote-2)^**

| ***Total cases (n)*** | **Non-severe**  **disease**  *60845* | **Severe disease**  *3198* | | **Critical disease**  *1129* | **Lethal disease**  970 | ***p-value*** |
| --- | --- | --- | --- | --- | --- | --- |
| ***Sociodemographic characteristics*** | | | | | | |
| *Women (%)* | 54 | 57.44 | | 44.20 | 53.81 | <0.001 |
| *Age(years) , mean (sd)* | 42.0 (19.4) | 62.9 (19.9) | | 67.3 (15.9) | 80.9 (10.9) | <0.001 |
| *Age range* | 0-103.7 | 0.1-103.7 | | 0-100.5 | 0-103.5 |  |
| *Age groups (%)* | | | | | | |
| 0-9 | 3,67 | 1,47 | | 0,8 | 0,1 | <0.001 |
| 10-19 | 11,72 | 2 | | 0,62 | 0 |  |
| 20-29 | 13,3 | 3,6 | | 0,89 | 0 |  |
| 30-39 | 18,78 | 7,29 | | 3,37 | 0,41 |  |
| 40-49 | 17,74 | 9,35 | | 7 | 0,93 |  |
| Under 50 | 65.21 | 23.7 | | 12.67 | 1.44 | <0.001 |
| 50-59 | 15.52 | 14.26 | | 13.91 | 3.09 |  |
| 60-69 | 11.77 | 21.04 | | 26.04 | 10.52 |  |
| 70-79 | 4.44 | 18.64 | | 24.62 | 22.27 |  |
| 80 and older | 3.07 | 22.36 | | 22.76 | 62.68 |  |
| ***Pre-COVID-19 comorbitity***  ***Charlson Index Score*** | | | | | | |
| *Charlson Index Score, mean (sd)* | 0.1 (0.4) | 0.5 (0.9) | | 0.7 (1.1) | 1 (1.1) | <0.001 |
| *Charlson index score range* | 0-9 | 0-8 | | 0-8 | 0-8 |  |
| *Charlson index score groups (%)* |  | | | | | |
| 0 | 92.26 | 69.98 | | 58.9 | 41.75 | <0.001 |
| 1–2 | 5.19 | 16.45 | | 20.54 | 30.52 |  |
| 3 or more | 2.55 | 13.57 | | 20.46 | 27.73 |  |
| *Charlson index components (%)* |  | | | | | |
| Acute Myocardial Infarction | 0.3 | 1.44 | | 3.9 | 2.47 | <0.001 |
| Congestive heart failure | 1.53 | 8.76 | | 11.34 | 20.62 | <0.001 |
| Peripheral Vascular Disease | 0.17 | 1.41 | | 2.66 | 2.06 | <0.001 |
| Cerebrovascular Disease | 0.53 | 3.28 | | 4.96 | 10.21 | <0.001 |
| Dementia | 0.47 | 2.28 | | 1.06 | 9.38 | <0.001 |
| Chronic pulmonary disease | 1.37 | 3.75 | | 5.31 | 4.95 | <0.001 |
| Rheumatologic disease | 0.47 | 1.25 | | 1.42 | 1.24 | <0.001 |
| Peptic Ulcer | 0.16 | 0.63 | | 0.89 | 1.13 | <0.001 |
| Chronic kidney disease | 0.12 | 1.31 | | 2.21 | 2.37 | <0.001 |
| Any malignancy | 1.37 | 6.54 | | 10.1 | 13.61 | <0.001 |
| AIDS/HIV | 0.02 | 0.06 | | 0.18 | 0 | <0.001 |
| Diabetes | 1.83 | 5.97 | | 8.68 | 7.84 | <0.001 |
| Cancer | 1.38 | 6.54 | | 10.1 | 13.7 | <0.001 |
| Liver Disease | 0.25 | 0.56 | | 0.71 | 0.52 | <0.001 |
| ***Other comorbidities (%)*** |  |  | |  |  |  |
| Obesity | 1.45 | 4.35 | | 8.86 | 3.61 | <0.001 |
| Hyperlipidaemia | 2.95 | 7.98 | | 12.84 | 8.25 | <0.001 |
| Hypertension | 15.23 | 46.53 | | 57.31 | 70.82 | <0.001 |
| ***Influenza and vaccination within 2 years before COVID-19*** | | | | | | |
| Influenza (during last 2 year) | 32.07 | 28.27 | 24.18 | | 16.19 | <0.001 |
| Flu vaccine (during last 2 year) (%) | 7.09 | 13.76 | | 12.75 | 18.87 | <0.001 |

1. Data are numbers (percentages) unless stated otherwise.

   [↑](#endnote-ref-2)
